# Hydrogel-Based Platforms for Wound Care: Integrated Strategies for Antimicrobial Delivery and Biofilm Management

**DOI:** 10.3390/gels12050398

**Published:** 2026-05-05

**Authors:** Gabriela Marcelina Mihai, Liviu Martin, Lucretiu Radu, Madalina Aldea, Sorin Nicolae Dinescu, Andrei Gresita, Mihai Ruscu, Ramona Constantina Vasile, Alexandra-Daniela Rotaru-Zavaleanu

**Affiliations:** 1 Department of Hygiene, University of Medicine and Pharmacy of Craiova, 2-4 Petru Rares Str., 200349 Craiova, Romania; 2 Faculty of Medical Care, Titu Maiorescu University, Văcărești Road, No. 187, 040051 Bucharest, Romania; liviu.martin@prof.utm.ro; 3 Department of Psychiatry, University of Medicine and Pharmacy of Craiova, 2-4 Petru Rares Str., 200349 Craiova, Romania; 4 Department of Epidemiology, University of Medicine and Pharmacy of Craiova, 2-4 Petru Rares Str., 200349 Craiova, Romania; sorin.dinescu@umfcv.ro (S.N.D.); mihai.ruscu@umfcv.ro (M.R.); alexandra.rotaru@umfcv.ro (A.-D.R.-Z.); 5 Department of Physiology, University of Medicine and Pharmacy of Craiova, 2-4 Petru Rares Str., 200349 Craiova, Romania; andrei.gresita@umfcv.ro

**Keywords:** hydrogel wound dressings, biofilm, antimicrobial delivery, chronic wounds, tissue regeneration, stimuli-responsive biomaterials, nanocomposite hydrogels, wound healing

## Abstract

Chronic wounds, diabetic foot ulcers, venous leg ulcers, and pressure injuries affect millions of patients worldwide and cost healthcare systems in the order of $150 billion annually, yet treatment options have changed less than the scale of the problem would suggest. Biofilm formation, documented in up to 78% of chronic wounds, is a central cause: bacteria embedded in extracellular polymeric matrices tolerate antimicrobial concentrations up to 1000-fold higher than planktonic cells and sustain a chronic inflammatory state that actively prevents tissue repair. Hydrogels, crosslinked polymer networks with high water content and tunable physicochemical properties, have been widely studied as platforms for addressing these challenges, though the distance between laboratory results and clinical practice remains considerable. While recent reviews have summarized hydrogel materials or antimicrobial strategies in isolation, this review takes a different approach: we treat infection, biofilm persistence, and impaired regeneration as interconnected processes that must be addressed simultaneously, and we examine biofilm management as a distinct therapeutic target rather than merely a subset of antimicrobial delivery. We analyze hydrogel-based wound care across three integrated domains: design principles (natural, synthetic, and hybrid polymer systems; crosslinking strategies; and stimuli-responsive architectures), antimicrobial delivery (silver, antibiotics, antimicrobial peptides, natural agents, and controlled-release systems), and biofilm management (nanoparticle-mediated disruption, enzymatic EPS degradation, photodynamic approaches, quorum-sensing inhibition, and anti-adhesive surface engineering). For each area, we critically evaluate what the preclinical evidence supports, where it falls short, and what would be needed to bridge the gap to clinical application. Translation remains uneven. Among the many FDA- and EMA-cleared hydrogel dressings currently in clinical use, most are simple moisture-retaining or silver-containing formulations, while the multifunctional systems that dominate the research literature are at earlier stages of development. We discuss the main translational priorities, including more predictive preclinical models, long-term nanomaterial safety, harmonized outcome reporting, manufacturing scalability, and health economic evidence, as areas where further work can meaningfully accelerate clinical adoption.

## 1. Introduction

**Figure 1:**
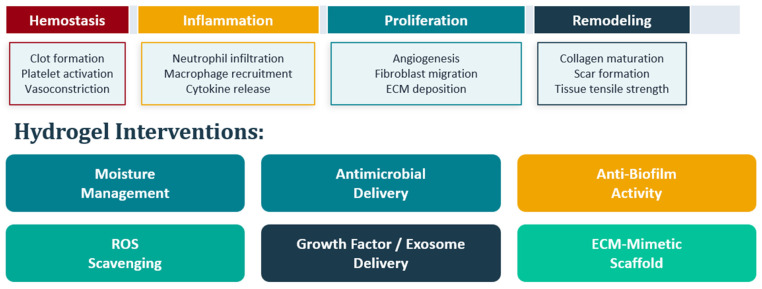
Schematic representation of the four overlapping phases of wound healing and the principal therapeutic roles of hydrogel-based dressings. Phases are depicted along a temporal axis (approximate durations vary by wound type, patient comorbidities, and infection status). Each hydrogel intervention (lower panel) may target one or more phases simultaneously, reflecting the multifunctional design strategy discussed throughout this review. Abbreviations: ECM, extracellular matrix; ROS, reactive oxygen species.

Wound healing follows a well-characterized sequence: hemostasis, inflammation, proliferation, remodeling. When it works, it resolves most acute injuries within weeks. When it does not work, the consequences are serious [1]. Chronic wounds, broadly defined as wounds that fail to proceed through this sequence within three months, affect an estimated 10.5 million Medicare beneficiaries in the United States alone, at an annual cost of approximately $22.5 billion [2]. Globally, wound care expenditure was estimated at approximately $148 billion in 2022, driven by aging populations, rising diabetes prevalence, and the belated recognition that chronic wounds in low- and middle-income countries represent a substantial and largely uncounted burden [2]. Figure 1 summarizes the four overlapping phases of wound healing and the corresponding therapeutic roles of hydrogel-based dressings addressed throughout this review.

The reasons wounds become chronic are multiple and often concurrent: diabetes impairs neutrophil function and microvascular perfusion; venous insufficiency creates sustained tissue edema and hypoxia; obesity increases mechanical stress on lower-extremity wounds; and immunosuppression compromises the inflammatory clearance phase. But across all etiologies, one factor recurs with striking consistency: the microbial biofilm. Malone et al. (2017) [3] identified biofilm in 78% of chronic wound samples, and while methodological differences between studies produce variable prevalence estimates, the clinical significance of biofilm in chronic wounds is now broadly recognized [3]. Biofilm-embedded bacteria tolerate antibiotic concentrations up to 1000-fold higher than their planktonic counterparts, resist phagocytic clearance, and maintain a state of perpetual low-grade inflammation that degrades granulation tissue and stalls the healing process [4]. Standard systemic antibiotic therapy is largely ineffective against this target, and topical antimicrobials, while better positioned, face diffusion barriers imposed by the extracellular polymeric matrix [5].

This is the context in which hydrogel-based wound dressings have been developed and studied. Hydrogels, three-dimensional crosslinked polymer networks with water contents of 70–99%, maintain the moist wound environment that Winter (1962) [6] demonstrated to be favorable for re-epithelialization, and their matrix can be engineered to carry and release antimicrobial agents, anti-inflammatory compounds, growth factors, or cells [6]. The tunability of polymer composition, crosslinking chemistry, and degradation kinetics provides, at least in theory, a platform adaptable to the heterogeneous demands of different wound types and healing stages [7].

The research literature on hydrogel wound dressings has grown rapidly: a PubMed search for “hydrogel wound healing” returns over 6000 results published since 2020. Much of this work is genuinely innovative in its materials science and biological design. The problem is not a lack of interesting formulations, but a lack of evidence that they work in patients. The vast majority of published studies report results from in vitro assays or acute wound models in young, healthy rodents, testing conditions that poorly predict performance in the actual clinical target: chronic, biofilm-infected wounds in elderly or diabetic patients with impaired perfusion and dysregulated immunity. Of the many hydrogel products with FDA or EMA clearance, most are simple amorphous gels or silver-containing dressings, while stimuli-responsive, nanocomposite, and exosome-loaded platforms remain predominantly at preclinical or early translational stages. Understanding what currently separates clinically adopted formulations from experimental ones is one of the central aims of this review.

The research literature on hydrogel-based wound dressings has expanded rapidly in recent years, and several reviews have contributed valuable perspectives on specific aspects of the field. Recent work has addressed antibacterial hydrogel formulations and their mechanisms of action [8], advancements in dressing materials including stimuli-responsive systems [9], smart dressings designed to combat antibiotic resistance, and hydrogel-based sensing and diagnostic platforms [10]. However, these reviews generally approach antimicrobial function and tissue regeneration as separate domains, which does not reflect the clinical reality that infection, biofilm persistence, and impaired healing are tightly interconnected processes in chronic wounds. Addressing one without the other is often insufficient. Furthermore, while existing reviews comprehensively catalog preclinical innovations, few critically examine why the translational gap between laboratory results and clinical practice remains so wide or identify the specific barriers, preclinical model limitations, long-term nanomaterial safety concerns, outcome standardization, manufacturing scalability, and health economic evidence that must be overcome for advanced hydrogel systems to reach patients. This review aims to fill that gap by providing an integrated, critically appraised analysis of hydrogel-based wound care across design principles, antimicrobial delivery, and biofilm management, with explicit attention being paid to the strength of evidence for each approach and the priorities for productive future research.

### Methodology and Scope of the Literature Reviewed

This article is presented as a critical narrative review rather than a systematic review; it does not follow PRISMA reporting standards, and no formal risk-of-bias appraisal or quantitative synthesis was undertaken. The relevant literature was identified through targeted searches of PubMed, Scopus, and Web of Science, supplemented by manual screening of the reference lists of recent reviews and by consultation of the FDA 510 (k), EMA, and ClinicalTrials.gov databases for regulatory and translational data. Search terms combined the keywords hydrogel, wound dressing, wound healing, chronic wound, biofilm, antimicrobial, antiseptic, silver, antimicrobial peptide, stimuli-responsive, and drug delivery, used singly and in Boolean combinations. The principal time frame considered was January 2015–October 2025, with priority given to publications from 2020 onward, which capture the recent expansion of the field; earlier seminal contributions were retained where they provide essential mechanistic or historical context. Eligible sources included peer-reviewed original research articles, narrative and systematic reviews, meta-analyses, clinical guidelines, and regulatory or clinical-trial registry records published in English. Conference abstracts, non-peer-reviewed preprints, and publications without an accessible English-language version were excluded. Selected references were prioritized for their relevance to the three integrated thematic domains structuring this review, hydrogel design principles, antimicrobial and antiseptic delivery, and biofilm management, with additional weight given to studies addressing chronic, biofilm-infected wound models or aspects of clinical translation.

## 2. Fundamentals of Hydrogel Design for Wound Applications

### 2.1. General Considerations

At their core, hydrogels are three-dimensional crosslinked polymer networks that absorb and retain large volumes of water, often several hundred times their dry weight, without dissolving [11,12]. This property makes them attractive for wound care, since a moist wound environment favors epithelial cell migration and granulation tissue formation [6,13]. However, moisture retention alone is a low bar: simple amorphous gels have provided this function adequately for decades, and the research effort invested in advanced hydrogel formulations is justified only if they deliver benefits beyond what existing products already achieve [14].

The appeal of hydrogels lies in tunability: stiffness, pore size, degradation rate, swelling behavior, and drug release kinetics can all be adjusted through polymer selection, crosslinking method, and formulation conditions [15]. This tunability is frequently invoked to argue that hydrogels can be tailored to the heterogeneous demands of different wound types. The extent to which this tunability has translated into demonstrated clinical benefit is more limited, and is a recurring theme throughout this review. Optimizing multiple parameters simultaneously requires systematic design-of-experiments approaches that are rarely employed; most published studies vary one or two parameters and report incremental improvements without establishing whether the resulting formulation is actually superior to simpler alternatives. More fundamentally, the vast majority of hydrogel formulations have been tested only in simplified in vitro settings or acute wound models in young, healthy rodents, and conditions that poorly predict performance in the actual clinical target: chronic, biofilm-infected wounds in elderly or diabetic patients with impaired perfusion and dysregulated immunity [16].

A large body of preclinical work describes formulations that perform well in laboratory models, although comparative clinical data against standard amorphous hydrogels or silver dressings remain limited. This gap partly explains the current translational bottleneck and motivates several of the priorities discussed throughout this review.

This gap between theoretical tunability and demonstrated clinical benefit is a central theme of this review. In the sections that follow, we examine the current evidence base relating hydrogel design parameters to wound healing outcomes and identify areas where further work is most needed.

### 2.2. Natural Polymer-Based Hydrogels

Natural polymers offer inherent biocompatibility and, in some cases, intrinsic bioactivity, but present challenges in reproducibility, mechanical performance, and degradation control that directly affect their utility as antimicrobial delivery platforms. Chitosan provides the only natural polymer with intrinsic broad-spectrum antimicrobial activity, mediated by electrostatic disruption of bacterial membranes through protonated amino groups [17].

However, this activity is pH-dependent (optimal below pH 6.5), which limits efficacy in the alkaline environment of many chronic wounds (pH 7.5–8.5) [18]. Mechanical brittleness typically requires blending with PVA or alginate, and the degree of deacetylation and molecular weight critically affect both antimicrobial potency and gelation behavior in ways that complicate cross-study comparisons [19,20].

Despite extensive preclinical investigation of chitosan hydrogels loaded with silver or antibiotics [21], clinical translation has followed a more selective path. Chitosan-based dressings are commercially available in several forms, primarily as hemostatic products used in trauma and surgical settings (HemCon, Tricol Biomedical, Portland, OR, USA; Celox, MedTrade Products, Crewe, UK; ChitoSAM, SAM Medical, Tualatin, OR, USA; ChitoGauze, Tricol Biomedical, Portland, OR, USA; Axiostat, Axio Biosolutions, Ahmedabad, India) and as gelling fiber or composite dressings for exudate management in chronic wounds (KytoCel, Aspen Medical Europe, Redditch, UK) [22,23]. Adoption in the specific context of biofilm-infected chronic wounds is more limited, and comparative clinical data against established alginate or foam dressings remain sparse [22,23].

Alginate offers mild gelation conditions (ionic crosslinking with Ca^2+^) favorable for encapsulating thermolabile antimicrobials and has an established clinical track record in wound care (Kaltostat, ConvaTec, Reading, UK; Sorbalgon, Hartmann, Heidenheim, Germany) [24,25,26]. The key limitations are mechanical instability in wound exudate (chelation-induced dissolution) and the absence of cell-adhesion motifs, which restricts regenerative applications unless it is RGD-modified [14,27].

Recent composite formulations incorporating bioactive glass or antimicrobial peptides show promise but lack clinical validation [28].

Hyaluronic acid (HA) uniquely participates in all wound healing phases, with molecular weight-dependent effects: high-MW HA is anti-inflammatory, while low-MW fragments (4–25 disaccharides) promote angiogenesis [29]. This creates both opportunity and complexity, intact HA in a hydrogel does not replicate the fragment-mediated signaling of natural wound healing, and enzymatic degradation kinetics are difficult to control [30].

Crosslinking options (EDC/NHS, thiol-ene, photo-crosslinking) each carry trade-offs in cytotoxicity and processing complexity [31]. High costs limit routine clinical use; existing HA wound products (Hyalofill, Anika Therapeutics, Bedford, MA, USA; Hyiodine, Contipro, Dolní Dobrouč, Czech Republic) target niche applications [32].

Bacterial cellulose (BC) provides exceptional water retention (up to 100× dry weight) and a nanofibrillar structure resembling native collagen, with favorable cell adhesion properties [33,34]. Limited clinical data exist, primarily from burn wound management [35], though large randomized controlled trials are lacking.

Non-degradability in vivo (humans lack cellulases) constrains applications requiring scaffold resorption [36,37].

Collagen and gelatin offer native cell-recognition sequences (RGD-like motifs) that support adhesion without modification [38]. GelMA (gelatin methacrylate) has become widely used due to tunable mechanical and degradation properties [39]. However, immunogenicity concerns with xenogeneic sources, rapid MMP-mediated degradation in chronic wounds, and batch variability limit reliability [40]. Clinical evidence for collagen dressings in chronic wounds remains low-certainty [41,42].

### 2.3. Synthetic Polymer-Based Hydrogels

Synthetic polymers offer reproducibility and mechanical tunability, but lack inherent bioactivity, requiring deliberate functionalization for cell interactions and antimicrobial performance (Table 1).

Poly(vinyl alcohol) (PVA) forms mechanically robust hydrogels through freeze–thaw cycling without chemical crosslinkers, avoiding cytotoxicity concerns [43]. Elastic modulus (1–10 kPa) can be tuned via molecular weight and cycle number [44]. The trade-off is limited intrinsic bioactivity: in its unmodified form, PVA shows minimal cell-adhesion or antimicrobial properties unless blended with bioactive materials (chitosan, alginate) or loaded with antimicrobial agents [12]. FDA-cleared PVA dressings exist (Hydrosorb, Hartmann, Heidenheim, Germany), but comparative clinical data supporting their superiority over established foam or hydrofiber alternatives for antimicrobial applications remain limited.

Poly(ethylene glycol) (PEG) resists protein adsorption and provides a “blank slate” for precise incorporation of adhesion peptides, growth factor binding sites, and degradable crosslinks [45]. This precision comes at a cost: every bioactive function must be engineered for, adding complexity and expenses that work against translation [46]. Multi-arm PEG hydrogels have shown promise in animal wound models when loaded with growth factors, but clinical adoption has been limited to surgical hemostasis (Coseal, Baxter International, Deerfield, IL, USA) rather than chronic wound management [47].

Poly(N-isopropylacrylamide) (PNIPAAm) exhibits thermoresponsive behavior (LCST ~32 °C) that has attracted interest for temperature-triggered drug release [48]. In practice, documented monomer cytotoxicity, abrupt phase transitions, and regulatory concerns have so far limited clinical translation [49,50]. To our knowledge, no PNIPAAm-based wound dressing has yet progressed beyond early preclinical studies.

### 2.4. Hybrid and Composite Approaches

Rather than choosing between natural and synthetic polymers, many recent studies combine them to offset their respective weaknesses. Typical strategies include interpenetrating polymer networks (IPNs), semi-IPNs, nanocomposite hydrogels (incorporating nanoparticles, nanofibers, or nanoclays), and sequential crosslinking methods [51].

The logic is sound: a chitosan/PVA blend can, in principle, combine the antimicrobial activity of chitosan with the mechanical strength of PVA. Alginate/GelMA composites can pair the easy gelation of alginate with the cell-adhesive properties of gelatin. Incorporating silica or laponite nanoparticles can improve mechanical properties while also serving as reservoirs for drug loading [52].

However, increased complexity brings its own problems. Each additional component introduces new variables (ratio, mixing order, crosslinking sequence, interaction effects) that must be optimized. Scale-up from laboratory batch sizes (typically 1–10 mL) to manufacturing quantities is non-trivial, and batch reproducibility, which is already a challenge with natural polymers, becomes harder as formulation complexity increases [53].

There is also a question of diminishing returns. A three- or four-component hydrogel may outperform simpler formulations in carefully controlled laboratory experiments, but the improvement margin often narrows substantially in animal models, where biological variability dominates. Whether the added manufacturing complexity and cost justify the incremental performance gain is a question that few studies address explicitly [54].

### 2.5. Crosslinking Strategies

The crosslinking method determines gel stability, mechanical properties, degradation behavior, and cytocompatibility. Three broad categories are used, as outlined below (Table 2).

Physical (non-covalent) crosslinking relies on hydrogen bonds, ionic interactions, hydrophobic associations, or crystallite formation. Alginate/Ca^2+^ and PVA freeze–thaw gels are the most common examples. Physical gels are generally easy to prepare under mild conditions, but tend to be mechanically weaker and less stable than covalent gels. They are also reversible, a feature that can be either advantageous (for injectable formulations) or problematic (for dressings that must maintain integrity over days) [55].

Chemical (covalent) crosslinking produces permanent networks with superior mechanical properties and stability. Common chemistries include glutaraldehyde treatment, carbodiimide coupling, photo-initiated radical polymerization, and various click chemistry approaches. The concern with chemical crosslinking is cytotoxicity, either from the crosslinker itself (glutaraldehyde is cytotoxic at low concentrations), from photoinitiator residues, or from unreacted functional groups [56].

Enzymatic crosslinking uses enzymes such as transglutaminase, horseradish peroxidase (HRP/H_2_O_2_), or tyrosinase to form covalent bonds under physiological conditions. This approach avoids the toxicity concerns of chemical crosslinkers and proceeds at neutral pH and 37 °C, making it compatible with cell encapsulation. The trade-offs are cost, limited availability for certain polymer backbones, and the need to carefully control enzyme-to-substrate ratios [57].

A meaningful comparison of crosslinking methods for wound care hydrogels would require standardized testing conditions, the same polymer, the same concentration, and comparable gel geometry, which is rarely available in the published literature. Most studies compare their chosen crosslinking method against a relatively weak control rather than against the best available alternative [58].

**Table 2:** Stimuli-responsive hydrogels for wound care applications.

Stimulus	Wound Trigger	Polymer Platform	Therapeutic Response	Development Stage
pH [59,60]	Infected: pH 5.5–6.5; Chronic: pH > 7.4	Chitosan, PAA, polybetaine, PLGA-PEG	Enhanced drug release; gelation; color-change sensing	Preclinical—advanced
Temperature [55,61]	Febrile/inflamed tissue > 37 °C	PNIPAAm, Pluronic F127, elastin-like polypeptides	Injectable → in situ gelation; accelerated release	Preclinical
ROS [62,63,64]	Elevated H_2_O_2_/superoxide in infected/inflamed tissue	Thioketal-PEG, boronic ester-PVA, selenide-crosslinked	ROS scavenging + triggered drug release; anti-inflammatory	Preclinical—early
Enzyme (MMPs/bacterial) [65]	Elevated MMP-2/9; bacterial hyaluronidase, lipase	MMP-cleavable peptide crosslinkers; HA substrates	On-demand release linked to infection severity	Research—proof of concept
Multi-stimuli [64,66,67]	Combined pH + ROS + enzyme activity	Hybrid platforms (e.g., HB/Ag/g, Hy-NO-Ag-Cip)	Precision adaptive therapy; reduced off-target effects	Preclinical—emerging

### 2.6. Physicochemical Properties and Biological Safety

The polymer choices and crosslinking strategies discussed above eventually come down to a handful of material properties that decide how a hydrogel actually behaves on a wound. It is worth bringing these together explicitly, since the link between formulation and function tends to be left implicit in most of the literature.

Mesh size is probably the single most important microstructural feature. It is set by crosslink density and polymer concentration, and it governs what can move through the network. Tight networks, with mesh sizes below about 10 nm, restrict the movement of larger molecules such as antimicrobial peptides or nanoparticles. This slows the release and extends the therapeutic window, but it can also keep the drug from reaching the interior of a mature biofilm. Looser networks above 50 nm allow for rapid diffusion at the familiar cost of burst release and premature depletion [68]. Pore architecture, meaning whether pores are interconnected or terminate as dead ends, also influences exudate handling and cell infiltration, although most studies still describe porosity only qualitatively through SEM images.

Mechanical properties matter for reasons that are sometimes overlooked in materials-focused papers. Soft tissue at the wound bed has a Young’s modulus somewhere in the range of 1–30 kPa, and hydrogels in that range support fibroblast activity and keratinocyte migration without triggering the stiffness responses associated with scarring [69]. A gel that is too soft collapses under exudate load or tears during dressing changes, while one that is too stiff conforms poorly to the wound surface and can raise peri-wound pressure. For dressings worn over several days, cohesive strength and fatigue resistance under repeated deformation end up mattering as much as the headline modulus value, and these are properties that most hydrogel papers simply do not report [70].

Degradation behavior has to fit the intended wear time. A gel used for exudate management typically needs to hold together for 24 h to several days, while a sustained-release platform may need to stay intact for one to two weeks before being replaced or cleared. Hydrolytic degradation, which is the dominant mechanism in PEG- or PLGA-containing systems, is relatively predictable. Enzymatic degradation is not. GelMA, hyaluronic acid, and collagen are cleaved by matrix metalloproteinases and hyaluronidases, and the activity of these enzymes in chronic wounds is both elevated and patient specific, which means that in vivo lifespan cannot reliably be extrapolated from the PBS studies at 37 °C that still dominate the field. The gap between laboratory degradation timelines and actual wound-bed kinetics remains an important consideration for translation and one of the areas where more in vivo characterization would strengthen the field [71].

Drug release kinetics follow a few familiar regimes—diffusion controlled, swelling controlled, and erosion controlled—and most practical formulations display some combination of these. A burst release of 30–50 percent of the payload within the first hour is the rule rather than the exception for physically entrapped antimicrobials, and it is probably the single biggest reason that systems advertised as sustained release often fail to hold their concentrations above MIC for more than a day [72]. Crosslinking chemistry can shift the release kinetics independently of network density, since residual functional groups along covalent backbones can form electrostatic or hydrogen bonding interactions with charged antimicrobials that slow diffusion [54]. When the crosslinks themselves are engineered to be cleaved by bacterial or host proteases, drug liberation becomes tied to infection severity—an idea that we return to in Section 3 [71].

Biological safety is an area where reporting practices vary across the hydrogel wound care literature and where more systematic characterization would further support clinical translation. Cytocompatibility is almost always reported, usually as fibroblast viability above 80 percent by MTT or Alamar Blue at 24 h, but that level of testing says little about what happens when a compromised tissue bed is repeatedly exposed to the same material over days or weeks. Residual crosslinkers and photoinitiators, including glutaraldehyde and lithium phenyl-2,4,6-trimethylbenzoylphosphinate, can leach in small amounts and affect local cell populations. Degradation products are rarely profiled for toxicity. Xenogeneic proteins from bovine collagen or fish gelatin carry real immunogenicity risk in a subset of patients. The long-term fate of nanomaterials embedded in the matrix, particularly silver, zinc oxide, and silica, becomes relevant once the polymer has cleared and the nanoparticles remain. Microbially sourced polymers such as bacterial cellulose or Streptococcus derived hyaluronic acid can carry endotoxin contamination that is not always caught by routine quality control [73,74,75,76]. ISO 10993 [77] testing covers most acute concerns, but does not really address the repeated exposure, compromised barrier scenarios that define chronic wound care.

Taking these five properties together, the common issue is that hydrogel performance is still evaluated almost entirely under standardized laboratory conditions, while the chronic wound is anything but standardized. Most published studies characterize either material properties or antimicrobial efficacy, and rarely both in a way that allows for a quantitative link to be drawn between formulation parameters and biological outcomes. Closing that structure function gap by running ex vivo wound bed characterizations and in vivo degradation and release studies in disease relevant models is a precondition for the structure–function understanding that rational hydrogel design ultimately requires.

## 3. Hydrogels as Platforms for Controlled Antiseptic and Antimicrobial Delivery

Antimicrobial delivery is the most extensively studied application of hydrogels in wound care, with hundreds of publications annually describing new formulations. For each major strategy discussed below, we summarize the current evidence base and outline what additional data would further support clinical translation. One recurring consideration across this literature is the difference in complexity between formulation design and efficacy testing: many studies report multi-component stimuli-responsive systems evaluated primarily through disc diffusion assays against planktonic laboratory strains, which provide a useful initial screen, but have limited predictive value for polymicrobial biofilm infections. Moving toward more representative testing models is a recognized next step for the field.

### 3.1. The Clinical Problem

Infection remains the most common complication in chronic wound management and the one most likely to derail an otherwise favorable healing trajectory. The microbiology of chronic wounds is complex: most harbor polymicrobial communities organized in biofilm, with *Staphylococcus aureus* (including MRSA), *Pseudomonas aeruginosa*, *Enterococcus* spp., and various anaerobes being the most frequently isolated species [78]. Topical antimicrobial treatment is generally preferred over systemic antibiotics for wound infections, both to achieve higher local drug concentrations and to limit systemic side effects and resistance selection [79].

Hydrogels are, in principle, well suited to serve as topical antimicrobial delivery vehicles. Their high water content allows for the dissolution and diffusion of hydrophilic drugs, their matrix can be engineered to modulate release kinetics, and the gel itself maintains wound moisture. In practice, the challenge lies in achieving antimicrobial concentrations high enough to penetrate biofilm and kill bacteria while keeping cytotoxicity to host cells (keratinocytes, fibroblasts, endothelial cells) at acceptable levels. This therapeutic window is narrower than is often acknowledged in the preclinical literature [80].

One consideration that recurs across the antimicrobial hydrogel literature is the difference in complexity between formulation design and efficacy testing. Many studies report multi-component stimuli-responsive systems evaluated primarily through disk diffusion assays against planktonic laboratory strains of *S. aureus* and *E. coli*. This testing format provides a useful initial screen, but has limited predictive value for the polymicrobial biofilm infections that characterize chronic wounds in practice. Moving toward more clinically representative testing models is a recognized priority for the field, and this caveat is worth keeping in mind when interpreting the preclinical results summarized in the following sections (Table 3).

The therapeutic window for hydrogel-delivered antimicrobials is determined not only by the intrinsic toxicity of the agent, but also by the hydrogel’s network architecture. Pore size and tortuosity control the rate at which antimicrobial molecules diffuse from the gel matrix into the wound bed and, critically, into the biofilm EPS. Hydrogels with excessively tight networks may retain antimicrobials too effectively, failing to achieve bactericidal concentrations at the biofilm interface, while overly porous gels release their payload too rapidly, resulting in high initial concentrations (with associated cytotoxicity) followed by sub-therapeutic levels that promote resistance selection [68,81]. Optimizing network architecture to match the physicochemical properties of the loaded antimicrobial, molecular weight, charge, and hydrophobicity is therefore a prerequisite for achieving the sustained, biofilm-penetrating release profiles that chronic wound management requires.

### 3.2. Silver-Based Systems

Silver has been used in wound care since antiquity, and silver-containing dressings (Aquacel Ag, ConvaTec, Reading, UK; Acticoat, Smith & Nephew, Watford, UK; Silvercel, 3M/Solventum, Maplewood, MN, USA) represent one of the few antimicrobial hydrogel-adjacent technologies with extensive clinical use. The antimicrobial mechanism involves release of Ag^+^ ions, which disrupt bacterial membrane integrity, inactivate respiratory enzymes, and interfere with DNA replication [82,83].

Silver nanoparticles (AgNPs) have become the dominant form studied in the hydrogel literature, largely because their size-dependent properties allow for some degree of control over ion release kinetics. Particles in the 10–30 nm range generally show higher antimicrobial activity per unit mass than larger particles due to the increased surface-area-to-volume ratio [84].

The problem with much of the AgNP-hydrogel literature is an imbalance between antimicrobial efficacy data and toxicity assessment. A substantial number of studies report zone-of-inhibition assays or planktonic kill curves against laboratory strains of *S. aureus* and *E. coli*, declare the system “biocompatible” on the basis of a single MTT assay at 24 h, and conclude with some variant of “promising for wound healing applications.” This pattern recurs so frequently that it has become difficult to distinguish genuinely useful formulations from incremental variations [85].

The toxicity concern is not hypothetical. AgNPs at concentrations above 10–25 μg/mL have been shown to be cytotoxic to human dermal fibroblasts and keratinocytes in multiple independent studies [68]. The therapeutic window, the ratio between the minimum inhibitory concentration for relevant pathogens and the concentration that impairs mammalian cell viability, is often narrow, particularly for MRSA and *Pseudomonas* biofilms, which require higher silver concentrations than planktonic bacteria [86].

There are also environmental and regulatory dimensions that the wound care literature largely ignores. Silver released from dressings enters wastewater systems, where it can disrupt microbial communities in treatment plants and accumulate in sewage sludge. The European Wound Management Association (EWMA) and several national guidelines now recommend limiting silver dressing use to two-week courses, with reassessment before continuation [83].

None of this means silver-based hydrogels are without value; they clearly have antimicrobial activity, and for short-term management of critically colonized wounds, the risk–benefit calculation may be favorable. But the literature would benefit from more honest acknowledgment of the limitations.

### 3.3. Antibiotic-Loaded Hydrogels

Loading conventional antibiotics into hydrogels offers the advantage of using drugs with well-characterized spectra of activity, established susceptibility testing protocols, and known pharmacokinetics. Gentamicin, ciprofloxacin, vancomycin, and metronidazole are among the most commonly studied [81].

The core technical challenge is controlling release kinetics. Most simple hydrogel formulations release their drug payload within hours to a few days through diffusion, which is often too fast to maintain therapeutic concentrations over the full treatment period. Burst release, where 50–80% of the loaded drug exits the gel in the first few hours, is a recurring problem in the literature and a genuine barrier to clinical utility [87].

Several strategies have been developed to slow release. Physical entrapment in denser networks, electrostatic binding between charged drugs and oppositely charged polymer chains, encapsulation in nanoparticles or liposomes that are then embedded in the hydrogel matrix, and covalent conjugation via hydrolyzable linkers all extend the release window, though each adds formulation complexity [88]. Drug-cyclodextrin inclusion complexes incorporated into hydrogels have shown some success in sustaining release of hydrophobic antibiotics over 7–14 days in vitro [89].

A broader concern with antibiotic-loaded wound dressings, hydrogel-based or otherwise, is the contribution to antimicrobial resistance. Sub-inhibitory antibiotic concentrations at the wound periphery, where drug has diffused away from the dressing center, can promote resistance development in wound flora [90]. This is not a theoretical risk: topical mupirocin resistance in MRSA has increased substantially since its widespread adoption for wound and nasal decolonization [91]. The same logic applies to any antibiotic delivered topically, and the wound care field has been slow to address this systematically.

For wounds with confirmed antibiotic-resistant organisms, conventional antibiotic-loaded hydrogels offer limited benefit, which is precisely the clinical scenario where new approaches are most needed.

The choice of crosslinking strategy has direct consequences for antibiotic release kinetics. Physically crosslinked hydrogels (e.g., alginate/Ca^2+^, PVA freeze–thaw) typically exhibit faster, diffusion-dominated release because the reversible nature of the crosslinks allows for network relaxation and swelling in wound exudate. Covalently crosslinked gels offer greater control: higher crosslink density reduces mesh size and slows diffusion, while the incorporation of hydrolytically labile crosslinks (e.g., ester bonds in PLGA-PEG systems) introduces a degradation-controlled release phase that can extend the therapeutic window from days to weeks [54,68]. For antibiotics with narrow therapeutic indices, such as aminoglycosides, this level of control is not optional—it is essential for maintaining efficacy without cytotoxicity.

**Table 3:** Antimicrobial agents incorporated into wound care hydrogels.

Agent Class	Key Agents	Target Pathogen	Release Mechanism	Representative Study	Key Limitation
Traditional Antibiotics	Ciprofloxacin, Gentamicin, Vancomycin, Tetracycline	Broad-spectrum; MRSA (vancomycin)	Diffusion; pH-triggered; degradation-controlled	[81,87]	Resistance risk; subtherapeutic levels at depth
Conventional Antiseptics	Povidone-iodine (PVP-I), Chlorhexidine (CHG)	Broad-spectrum bacteria, fungi, viruses	Slow sustained release; encapsulation	[10,79]	Cytotoxicity at high conc.; thyroid effects (iodine)
Silver Nanoparticles (AgNPs)	Ag^0^/Ag^+^, ultrasmall (<10 nm)	MRSA, *P. aeruginosa*, biofilm	Ion release + direct membrane disruption	[66,67]	Nanoparticle cytotoxicity; bioaccumulation
Metal Oxide NPs (ZnO, CuO)	ZnO, CuO, CeO_2_ nanoparticles	Broad-spectrum; angiogenesis promotion (Cu)	ROS generation; ion release; membrane disruption	[21,73]	Dose-dependent cytotoxicity; regulatory gap
Natural/AMP	Curcumin, Quercetin, AMPs (LL-37, nisin)	Broad; anti-biofilm; anti-inflammatory	Surface immobilization; diffusion; nano-encapsulation	[32,71,81]	Poor solubility (curcumin); stability; cost (AMP)

### 3.4. Antimicrobial Peptides

Antimicrobial peptides (AMPs) have generated substantial academic interest as alternatives to conventional antibiotics, primarily because their mechanism of action—membrane disruption through electrostatic interaction with the bacterial lipid bilayer—is thought to be less susceptible to conventional resistance mechanisms [92]. Hundreds of natural and synthetic AMPs have been identified, with LL-37, defensins, magainins, and various engineered variants being the most studied in wound care contexts [93].

Incorporating AMPs into hydrogels addresses one of their key limitations: rapid degradation by wound proteases. Free AMPs applied topically to chronic wounds are typically inactivated within minutes to hours by the high protease activity characteristic of the chronic wound environment. Hydrogel encapsulation or covalent tethering to the polymer matrix can extend AMPs’ stability and activity, at least in vitro [94].

The gap between the in vitro literature and clinical reality is wide, however, and needs to be stated plainly. As of early 2025, no AMP-loaded hydrogel has been approved for clinical wound care. The obstacles are not purely scientific; they are also economic and regulatory. AMPs are expensive to produce at pharmaceutical grade (solid-phase peptide synthesis is inherently costly for peptides longer than ~15 amino acids), batch-to-batch consistency is more difficult to achieve than for small-molecule drugs, and the regulatory pathway for a combination product (hydrogel device + peptide drug) is more complex than for either component alone [95].

There is also emerging evidence that the assumption of low resistance potential may be overly optimistic. Several studies have demonstrated that bacteria can develop resistance to AMPs through modifications of membrane lipid composition, upregulation of efflux pumps, and production of extracellular proteases that degrade the peptides [96]. The clinical significance of these mechanisms is unclear, but they weaken the argument that AMPs are fundamentally resistance-proof.

Research in this area continues to be active and worthwhile, but the timeline to clinical products is likely measured in decades rather than years.

### 3.5. Natural Antimicrobial Agents

A parallel line of research has explored loading hydrogels with plant-derived or other natural antimicrobial compounds: honey, curcumin, essential oils (tea tree oil, thymol, carvacrol), propolis, and various polyphenols [97].

Honey deserves a separate mention because it has the strongest clinical evidence base among the natural antimicrobials for wound care. Medical-grade manuka honey (Medihoney, Comvita/Derma Sciences, Princeton, NJ, USA; Revamil, Bfactory Health Products, Rhenen, The Netherlands) is already used clinically, and its antimicrobial activity, attributed to hydrogen peroxide generation, methylglyoxal content, low pH, and high osmolarity, is well documented against a broad range of wound pathogens, including MRSA [98]. Incorporating honey into hydrogel matrices has been explored by several groups, with the main technical challenge being that honey’s high sugar content and low pH can interfere with the gelation of certain polymers [99].

Curcumin has attracted outsized attention relative to its practical utility. While it has documented anti-inflammatory and modest antimicrobial properties in vitro, its extremely low aqueous solubility, rapid degradation, and poor bioavailability have limited every clinical application attempted to date, not just in wound care, but across oncology, neurology, and gastroenterology [100]. Nanoencapsulation within hydrogels can improve stability and local delivery, but the fundamental question of whether curcumin concentrations achievable at the wound site are sufficient for clinically meaningful antimicrobial or anti-inflammatory effects has not been convincingly answered [101].

Essential oils face a different set of challenges: variable composition depending on plant source, season, and extraction method; volatility that leads to rapid loss of activity; and a dose-dependent cytotoxicity profile that overlaps uncomfortably with the antimicrobial concentration range [102]. Encapsulation in nanoparticles or cyclodextrins before incorporation into hydrogels can mitigate volatility, but adds cost and complexity.

The natural antimicrobial field suffers from a reproducibility problem. Many studies use crude extracts rather than purified compounds, report antimicrobial activity against only one or two reference strains, and do not perform rigorous cytotoxicity testing. Until standardized extract preparations and testing protocols become the norm, it will remain difficult to identify which natural agents, if any, offer genuine advantages over established antimicrobials [71].

### 3.6. Controlled and Stimuli-Responsive Release

The ideal antimicrobial wound dressing would release its payload in response to infection rather than continuously, delivering drugs when bacterial burden is high and conserving it when the wound heals normally. This concept has driven research into stimuli-responsive hydrogels that release antimicrobials in response to specific wound environment signals [59].

pH-responsive systems exploit the fact that infected wounds tend to have a more alkaline pH (7.5–8.5) than healing wounds (5.5–6.5). Hydrogels with pH-sensitive bonds (Schiff bases, boronate esters, acetal linkages) can be engineered to degrade faster and release drugs at elevated pH [60]. The concept is elegant in theory. In practice, pH variation within a single wound can be substantial, the wound center, edge, and peri-wound skin may differ by 1–2 pH units, and the pH “trigger” may not be specific enough to distinguish infection from normal inflammatory fluctuations [103].

Enzyme-responsive systems target specific enzymes overexpressed in infected or inflamed wounds, typically bacterial lipases, gelatinases, or host-derived MMPs and hyaluronidases. Crosslinks or drug–polymer bonds that are cleaved by these enzymes release drug proportionally to enzyme activity [65]. This is a more specific trigger than pH, but enzyme concentrations vary enormously between patients and wound types, making dose prediction difficult.

Thermoresponsive systems based on PNIPAAm or similar polymers can release drug upon warming to body temperature. The clinical relevance remains uncertain: wound temperature typically does not vary by a margin sufficient to serve as a reliable trigger during infection, and the relatively constant warmth of a covered wound may produce sustained rather than triggered release under most conditions [61].

Reactive oxygen species (ROS)-responsive systems are among the more recent additions. Infected and inflamed wounds generate elevated levels of ROS (H_2_O_2_, superoxide, hydroxyl radicals), which can cleave thioether, selenide, or boronic ester bonds incorporated into the hydrogel network [62]. Early in vitro results are encouraging, but ROS levels in wounds are notoriously variable and difficult to measure accurately, and the long-term stability of ROS-sensitive hydrogels during storage is a concern that few studies have addressed [63].

Across all stimuli-responsive systems, the underlying principle is the same: the hydrogel’s response to environmental triggers is encoded in its molecular architecture. pH-responsive release requires incorporation of ionizable groups (carboxylic acids, amines) whose protonation state changes across the wound pH range. ROS-responsive release requires cleavable bonds (thioethers, boronic esters) positioned within the crosslink structure such that their scission increases mesh size or liberates drug conjugates. Enzyme-responsive release requires peptide sequences recognized by wound-relevant proteases (MMPs, bacterial lipases) to be incorporated as crosslinks or drug-polymer tethers. In each case, the design parameter (functional group identity, crosslink chemistry, peptide sequence) determines the stimulus specificity, response threshold, and release kinetics. This tight coupling between molecular design and functional performance is what makes stimuli-responsive hydrogels conceptually powerful, but also what makes their optimization challenging, since each parameter must be tuned to the heterogeneous and variable conditions of the chronic wound environment [65].

### 3.7. Combination and Synergistic Approaches

An increasingly common strategy is to combine two or more antimicrobial agents within a single hydrogel, for example, silver nanoparticles with an antibiotic or an AMP with a natural antimicrobial compound. The rationale is to broaden the antimicrobial spectrum, reduce the required concentration of each individual agent (thereby reducing toxicity), and potentially achieve synergistic killing [104].

Synergy is frequently claimed, but less frequently demonstrated rigorously. True synergy requires formal testing, typically checkerboard assays yielding fractional inhibitory concentration (FIC) indices below 0.5, or time-kill studies showing ≥ 2 log_10_ greater kill than the most active single agent. Many studies use the term “synergy” loosely, based on the observation that two agents together work better than either alone, without performing the quantitative analysis needed to distinguish synergy from simple additivity [105].

When genuine synergy has been demonstrated, for example, between silver and certain β-lactam antibiotics against MRSA or between chitosan and essential oil components against Gram-negative bacteria, the results can be clinically relevant because they allow for effective concentrations to be lowered into a less cytotoxic range [106]. However, synergy observed in planktonic cultures does not always translate to biofilm conditions where penetration barriers and metabolic heterogeneity introduce additional variables [107].

Multi-agent hydrogels also complicate manufacturing and quality control. Each active component requires separate stability testing, release characterization, and interaction studies. Regulatory agencies evaluate combination products more stringently than single-agent devices, which extends the approval timeline and increases development costs [108].

## 4. Biofilm Management and Anti-Biofilm Strategies Using Hydrogels

Biofilm management represents a fundamentally different challenge from antimicrobial delivery against planktonic bacteria, yet this distinction is frequently blurred in the wound care literature. Many studies claim “anti-biofilm” activity based on assays that measure only planktonic killing or early biofilm prevention without demonstrating disruption of established, mature biofilms. In this section, we distinguish carefully between these endpoints and assess the evidence for genuine biofilm disruption strategies.

### 4.1. Why Biofilms Matter and Why They Are Difficult to Treat

Most bacteria in chronic wounds do not float freely. They live in biofilms, structured communities encased in a self-produced matrix of polysaccharides, proteins, extracellular DNA, and lipids that collectively form what is referred to as the extracellular polymeric substance (EPS). Malone et al. (2017) found biofilm in 78% of chronic wound samples, and while the exact prevalence depends on the detection method used (microscopy tends to yield higher estimates than culture-based approaches), there is broad agreement that biofilm is the rule rather than the exception in wounds that fail to heal [3].

The clinical importance of biofilm is not simply that bacteria are present, it is that biofilm-resident bacteria behave fundamentally differently from planktonic cells. Antibiotic tolerance increases by as much as 1000-fold, driven not by acquired resistance genes, but by a combination of restricted drug diffusion through the EPS, reduced metabolic activity in deeper biofilm layers, and the presence of phenotypically dormant “persister” cells that survive antibiotic exposure by virtue of being metabolically quiescent rather than genetically resistant [109]. This is a critical distinction. It means that susceptibility testing performed on planktonic isolates, which is what clinical microbiology laboratories routinely provide, may be irrelevant to the actual situation in the wound.

The downstream effects on wound healing are well characterized. Biofilm maintains a self-perpetuating inflammatory state: the immune system recognizes the bacterial presence and mounts a response (elevated TNF-α, IL-1β, IL-6, MMPs, reactive oxygen species), but cannot clear the infection because the EPS shields bacteria from phagocytosis and opsonization. The result is persistent tissue destruction: granulation tissue is degraded as fast as it forms, angiogenesis is impaired, and the wound stalls in the inflammatory phase [110]. Adding to this, microbial metabolism within biofilms creates local hypoxia, which reduces the efficacy of both oxygen-dependent neutrophil killing and systemically administered antibiotics that require aerobic conditions for uptake.

The polymicrobial character of most wound biofilms adds another layer of complexity. *S. aureus* and *P. aeruginosa* are the species most frequently discussed, but clinical wound biofilms typically contain *Enterococcus*, *Enterobacter*, various anaerobes, and sometimes *Candida* species. These organisms do not simply coexist; interspecies interactions within the biofilm can enhance collective resistance. *P. aeruginosa*, for instance, produces alginate that physically shields neighboring *S. aureus* cells from antibiotic exposure, while staphylococcal biofilm components can protect *Pseudomonas* from host immune clearance [78]. Modeling these interactions in vitro remains challenging, and most published anti-biofilm studies test against single-species biofilms that do not capture this complexity.

Systemic antibiotics are generally ineffective against wound biofilms, not because the organisms are genetically resistant (though they may be), but because the drug concentrations achievable in wound tissue rarely exceed the elevated tolerance thresholds of biofilm-embedded bacteria. Topical antimicrobials penetrate better, but still face the EPS diffusion barrier. This is where hydrogel-based approaches enter the picture: not as a silver bullet, but as a delivery platform that can potentially address some of the specific challenges that make biofilm treatment so difficult.

### 4.2. Hydrogel-Mediated Biofilm Disruption

The basic case for hydrogels in anti-biofilm therapy rests on a few practical properties. They conform to irregular wound surfaces, ensuring contact with the biofilm–tissue interface. Their water-rich matrix is compatible with hydrophilic antimicrobials and allows for diffusion-based drug release. And the gel network itself can be engineered to interact with biofilm components, either passively (through sustained drug delivery) or actively (through incorporation of enzymes, nanoparticles, or responsive elements).

That said, “disruption of biofilm” is used loosely in the literature and deserves clarification. True biofilm disruption means degradation or destabilization of the EPS matrix such that the embedded bacteria become accessible to antimicrobial agents or immune clearance. This is different from simply killing planktonic bacteria released from a biofilm surface, which many conventional antimicrobials can do without affecting the biofilm itself.

#### 4.2.1. Nanoparticle-Loaded Systems

Silver nanoparticles remain the most studied anti-biofilm agent in hydrogel platforms. The work by Haidari et al. (2021) [111] is among the more rigorous in this space. Their AgNP hydrogel (200 μg/g silver) was tested against established *S. aureus* (Xen 29) biofilms, not just planktonic cultures, using both confocal microscopy (SYTO9/propidium iodide live/dead staining) and in vivo wound models. The confocal data showed genuine structural disruption of the biofilm, not merely surface killing, with a clear shift from viable to dead bacteria throughout the biofilm depth. In the wound model, AgNP hydrogel achieved 46% closure versus 20% for silver sulfadiazine, which is notable because silver sulfadiazine killed planktonic bacteria effectively, but left the biofilm architecture largely intact [111].

This study illustrates a point worth emphasizing: planktonic killing and biofilm disruption are different endpoints, and a product that performs well on one may fail at the other. Silver sulfadiazine has been a standard topical antimicrobial for decades and works adequately against free-floating bacteria, but it does not penetrate or disrupt established biofilms effectively. Nanoparticulate silver can do this, at least in some formulations, because particles below ~10 nm approach the EPS pore dimensions and can diffuse into the matrix [112]. Whether this translates to clinical superiority in human chronic wounds has not been tested in adequately powered randomized trials.

Aggregation is a practical concern. AgNPs tend to cluster in biological environments, which increases the effective particle size and reduces biofilm penetration. Hydrogel encapsulation partially addresses this by maintaining nanoparticle dispersion, but long-term stability during storage and upon contact with wound exudate has not been well characterized for most formulations.

#### 4.2.2. Enzyme-Mediated Dispersal

A conceptually different approach is to attack the biofilm matrix directly using enzymes that degrade its structural components. The logic is straightforward: if the EPS is what protects biofilm bacteria, then degrading the EPS should expose them to conventional antimicrobials or immune clearance.

Lysostaphin is one of the better-studied examples, a zinc metalloendopeptidase that cleaves the pentaglycine cross-bridges specific to *S. aureus* cell walls. Nithya et al. incorporated lysostaphin into a chitosan hydrogel and reported 95% bacterial lysis within 15 min in vitro, with acceptable cytotoxicity [113]. The specificity for *S. aureus* is both an advantage (low off-target effects) and a limitation (irrelevant for *Pseudomonas* or mixed-species biofilms).

Other enzymes under investigation include dispersin B (targets poly-β-1,6-N-acetylglucosamine, a key adhesin in staphylococcal biofilms), α-amylase (degrades polysaccharide EPS), and DNase I (cleaves the extracellular DNA scaffold present in many biofilms). The combination of an EPS-degrading enzyme with a conventional antibiotic, a “disrupt then kill” sequence, is appealing because enzymatic matrix degradation can restore antibiotic sensitivity in bacteria that were previously tolerant due to poor drug penetration [114].

Two practical issues limit this approach. First, enzyme stability in the wound environment is uncertain: wound proteases may degrade the therapeutic enzyme before it reaches the biofilm. Second, most of the evidence is from single-species in vitro biofilm models, and the EPS composition of clinical polymicrobial biofilms is considerably more heterogeneous and variable compared to what any single enzyme can address.

#### 4.2.3. Photodynamic and Photothermal Approaches

Photoactive hydrogels combine a photosensitizer or photothermal agent with light activation to generate localized antimicrobial effects. A methylene blue-loaded hydrogel (HG1MB1) demonstrated better penetration into biofilm depth than free methylene blue in solution, likely because the gel maintained prolonged contact and facilitated diffusion into the hydrated EPS [115]. Light activation generated reactive oxygen species that damaged both bacterial membranes and EPS components. Copper nanoparticle-embedded hydrogels with photothermal capability take a different approach: near-infrared irradiation generates localized heat that, combined with Cu^2+^ ion release, disrupts biofilm integrity [116].

These systems face an obvious practical constraint: they require an external light source, which limits their use to supervised clinical settings and cannot provide continuous anti-biofilm activity between dressing changes. For superficial, accessible wounds, this may be acceptable. For deep or cavity wounds, light delivery is problematic. The clinical workflow—apply hydrogel, position light source, irradiate for a specified duration, and remove—is also more complex than simply applying a dressing, which affects both the cost and patient compliance.

### 4.3. Preventing Biofilm Formation

Disrupting an established biofilm is difficult. Preventing bacteria from forming one in the first place is, at least in theory, a more tractable problem.

#### 4.3.1. Anti-Adhesive Surfaces

Hyaluronic acid coatings on wound dressings create a hydration layer that physically interferes with bacterial adhesion to the dressing surface. Kim et al. (2024) showed that HA-based dressings reduced both wound-dressing adhesion (preventing re-injury during dressing changes) and bacterial colonization [117]. Zhang et al. developed a self-healing HA hydrogel using dynamic covalent bonds (aldehyde HA crosslinked with DTP and quaternized chitosan), delivered through a dual-syringe system [118]. The self-healing property is practically relevant: wound dressings are subject to mechanical stress during patient movement, and a gel that can reform after disruption maintains its barrier function better than one that cracks and fragments.

Zwitterionic polymers, poly(sulfobetaine methacrylate) and poly(carboxybetaine methacrylate), offer a complementary strategy. Their strong hydration through electrostatic interactions with water creates a surface that resists protein adsorption and bacterial attachment. As an outer layer in a multilayer hydrogel dressing, a zwitterionic coating can minimize bacterial colonization of the dressing itself while inner layers handle antimicrobial delivery or tissue support [119].

The limitation of anti-adhesive approaches is that they protect the dressing surface but do not directly address the biofilm already present on the wound bed. They are best understood as adjunctive measures useful for preventing dressing-associated recontamination, but insufficient as standalone anti-biofilm therapy.

#### 4.3.2. Contact-Killing AMP Surfaces

Covalent attachment of antimicrobial peptides to hydrogel surfaces creates a non-releasing bactericidal surface; bacteria that land on it are killed on contact. AMP-functionalized Pluronic F127 hydrogels (using RRP9W4N peptide conjugated via EDC/NHS chemistry) showed activity against *S. epidermidis*, *S. aureus*, *P. aeruginosa*, MRSA, and MDR *E. coli* over 24 h, without detectable mammalian cell toxicity [120].

A subsequent study identified synergistic AMP combinations: co-functionalization with indolicidin (3.1 μM) and P10 (12.5 μM) at half their individual effective concentrations killed MRSA effectively [121]. This is a meaningful finding because it addresses the cost barrier, AMPs are expensive to synthesize, and halving the required amount improves the economics considerably.

Whether tethered AMPs retain long-term activity in the wound environment, where protease-rich exudate could cleave the peptide from the surface or degrade it in place, remains an open question. Most studies evaluate activity at 24 h; data beyond a few days are scarce.

### 4.4. Combination and Multi-Modal Strategies

Single-mechanism approaches to biofilm management have inherent limitations. Biofilms are adaptive systems: they can upregulate EPS production in response to antimicrobial stress, recruit resistant species from the wound microenvironment, and reform rapidly after incomplete disruption. This biological reality has pushed the field toward multi-modal strategies that attack biofilm through complementary mechanisms simultaneously.

#### 4.4.1. Quorum Sensing Inhibition

Quorum sensing (QS), the chemical communication system through which bacteria coordinate biofilm formation, virulence factor production, and collective behavior, is an attractive target precisely because it does not kill bacteria directly. By disrupting communication without imposing lethal selective pressure, QS inhibition theoretically reduces the risk of resistance emergence [122].

An ROS-scavenging hydrogel loaded with hyperbranched poly-L-lysine (HBPL) illustrates the dual-action concept [64]. HBPL blocked the *S. aureus* agr quorum sensing pathway, reducing virulence factor production (hemolysins, proteases), while its cationic charge simultaneously provided direct membrane-disruptive bactericidal activity. The hydrogel’s thioketone and disulfide crosslinks scavenged reactive oxygen species, addressing the inflammatory component of the chronic wound. In a full-thickness MRSA wound model, this system produced scar-free healing, a result that, if reproducible, would be clinically significant.

Engineered lactonases such as SsoPox-W263I have shown efficacy against *P. aeruginosa* QS specifically, inhibiting virulence factor expression and biofilm formation in isolates from diabetic foot ulcers [123]. Integration of these enzymes into hydrogel platforms for sustained local delivery is a logical next step, though formulation challenges (enzyme stability, controlled release kinetics, sterilization compatibility) have not been fully resolved.

A note of caution on QS inhibition: while the concept is appealing, bacteria possess redundant communication systems, and blocking one QS pathway may not prevent biofilm formation if alternative pathways compensate. The agr system in *S. aureus* is only one of several regulatory networks governing biofilm behavior, and *P. aeruginosa* has at least three interconnected QS systems (las, rhl, PQS). Truly effective QS inhibition may require targeting multiple pathways simultaneously, which increases complexity.

#### 4.4.2. Multi-Agent Platforms

The triple-agent Hy-NO-Ag-Cip hydrogel exemplifies the multi-modal concept in practice: nitric oxide disperses the EPS and sensitizes biofilm bacteria, silver nanoparticles provide broad-spectrum killing through multiple mechanisms (membrane disruption, ROS generation, protein inactivation), and ciprofloxacin adds targeted antibiotic activity. At 2 × MIC, this combination reduced established *P. aeruginosa* biofilm by 65% and achieved 90% wound closure by day 11 in a rat burn wound model [66].

These numbers warrant careful interpretation. A 65% reduction in biofilm biomass, while substantial, means that 35% of the biofilm survived, and given biofilm’s capacity for rapid regrowth from surviving cells, this may not be sufficient for clinical resolution without repeated applications or adjunctive debridement. The 90% wound closure figure is from an acute burn wound model in young, healthy rats; chronic wounds in diabetic or elderly patients present a far more hostile healing environment. Extrapolating these results to clinical practice requires considerable caution.

#### 4.4.3. Stimuli-Responsive Anti-Biofilm Systems

pH-responsive hydrogels that increase drug release at alkaline pH (>7.4, characteristic of infected wounds) provide a form of self-regulating treatment: drug delivery intensifies when infection is likely present and decreases as the wound heals and pH normalizes. Haidari et al. demonstrated an AgNP hydrogel that limited Ag^+^ release at acidic pH but achieved > 95% pathogen elimination at alkaline pH, which is conceptually elegant [67].

In practice, pH is an imperfect proxy for infection. Wound pH is influenced by factors beyond bacterial presence, ischemia, necrotic tissue, dressing composition, exudate volume, and varies spatially within a single wound. A hydrogel that responds primarily to pH may release drugs over healthy wound edges (where pH can be elevated for non-infectious reasons) while under-dosing areas of heavy biofilm burden in wound recesses.

Enzyme-responsive and ROS-responsive systems face analogous specificity concerns: the triggering molecules (bacterial enzymes, reactive oxygen species) are also produced by host inflammatory processes, blurring the distinction between “infection-responsive” and “inflammation-responsive” drug release.

#### 4.4.4. Magnetic and Ultrasound-Enhanced Penetration

**Figure 2:**
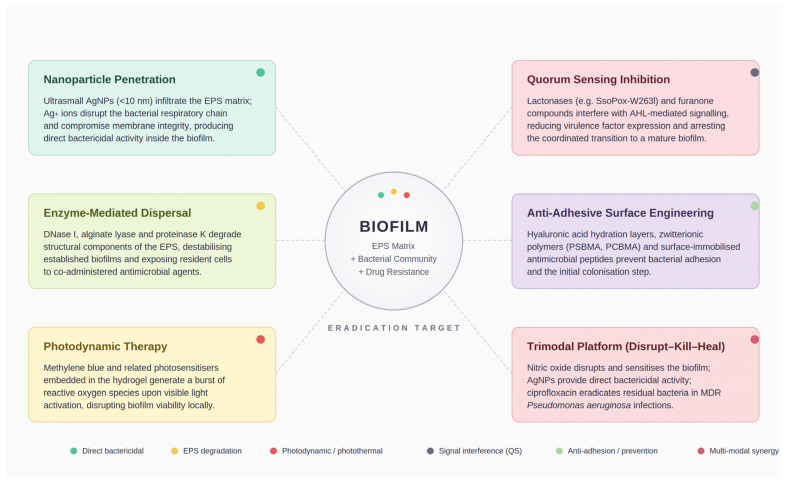
Schematic overview of the six anti-biofilm mechanisms employed by hydrogel-based wound dressings targeting the central pathological entity, the bacterial biofilm. Left column: Mechanisms of active biofilm disruption and eradication. Right column: Strategies targeting biofilm initiation, signaling, and multimodal eradication. Central: Representation of biofilm structure (EPS matrix + bacterial community). Abbreviations: EPS—extracellular polymeric substance; AgNP—silver nanoparticle; QS—quorum sensing; AHL—acyl-homoserine lactone; MDR—multidrug resistant.

More exotic approaches include Ag/Fe_3_O_4_ bimetallic nanocomposites in hydrogels, where external magnetic fields drive nanoparticle penetration into the biofilm matrix and ultrasound-responsive alginate hydrogels loaded with neomycin, bacitracin, and polymyxin B [124]. The latter claims real-time ultrasound monitoring of bacterial colony formation within the wound, a theranostic concept (simultaneous diagnosis and treatment) that is intellectually compelling, but would require specialized equipment at the point of care (Figure 2).

These technologies are worth mentioning for completeness, but are far from clinical application. The infrastructure requirements (magnetic field generators, therapeutic ultrasound equipment, and trained operators) are incompatible with outpatient wound care settings, where the vast majority of chronic wound management occurs.

### 4.5. Current Limitations in the Field

It would be incomplete to discuss anti-biofilm hydrogels without addressing a systemic problem in the preclinical literature. The majority of published studies evaluate their hydrogels against either planktonic bacteria, young single-species biofilms grown on plastic or glass surfaces for 24–48 h, or, at best, single-species biofilms in acute wound models in young, healthy rodents. None of these adequately represent the clinical target: mature, polymicrobial biofilms embedded in devitalized tissue in a wound bed with impaired perfusion and a dysregulated immune response.

The gap is not small. A 24 h *S. aureus* biofilm on a polystyrene well plate is structurally and metabolically different from a months-old polymicrobial biofilm in a diabetic foot ulcer. Systems that show 95% or 99% reductions in the former may show substantially reduced efficacy against the latter, and direct data bridging this gap remain scarce.

Until the field adopts more clinically relevant testing models, mature polymicrobial biofilms on ex vivo wound substrates, chronic wound animal models with established infections (e.g., delayed-healing diabetic mouse models or porcine chronic wound models), and properly designed clinical trials with biofilm-specific endpoints, the gap between laboratory results and clinical utility will persist. This is not a call for pessimism, but for methodological rigor: the biological tools described in this chapter may genuinely work, but proving it requires better experiments than most of the current literature provides.

## 5. From Bench to Bedside: Current Status of Hydrogel Wound Dressings

### 5.1. The Preclinical Pipeline and Its Limitations

Every hydrogel wound dressing starts in the lab, and the path from there to a patient’s wound is long, expensive, and littered with failures. It helps to be honest about what each stage of evaluation actually tells us and what it does not.

In vitro testing is where most published studies stop. The standard package, an MTT or live/dead assay on fibroblasts or keratinocytes, a disk diffusion or planktonic kill assay against *S. aureus* and *E. coli*, perhaps crystal violet biofilm quantification or confocal imaging, is necessary as a first screen, but its predictive value for clinical performance is limited. Monospecies biofilms grown on polystyrene for 24 h do not resemble the polymicrobial, matrix-rich, immune-active biofilms in a three-month-old diabetic foot ulcer. The Lubbock chronic wound biofilm model, which co-cultures *S. aureus*, *P. aeruginosa*, and *E. faecalis* in wound-simulating media, is a step in the right direction [4], but it still lacks immune cell interactions, tissue architecture, and the variable perfusion that characterizes real wounds.

#### 5.1.1. Advanced In Vitro Wound Models

Recognition of these limitations has driven the development of more sophisticated in vitro platforms that better approximate the chronic wound environment, though their adoption in hydrogel evaluation remains limited.

Multi-species biofilm models address the polymicrobial nature of clinical wound infections. Beyond the Lubbock model, continuous-flow systems such as drip-flow reactors and CDC biofilm reactors generate mature, structured biofilms under shear conditions that more closely resemble in vivo biofilm architecture than static well-plate cultures [125]. Colony–biofilm models on semi-permeable membranes allow for nutrient gradients and spatial heterogeneity that produce metabolically stratified communities with dormant persister cells in deeper layers [126]. Co-culture systems pairing *S. aureus* with *P. aeruginosa* have revealed interspecies interactions, including cross-protection and metabolic cooperation, that significantly increase antimicrobial tolerance compared to single-species biofilms [68]. However, even these advanced biofilm models typically lack host cells and immune components, limiting their ability to predict the inflammatory modulation that is central to chronic wound pathology.

Three-dimensional tissue-engineered skin constructs incorporate dermal fibroblasts in collagen or fibrin matrices overlaid with stratified keratinocyte layers, creating full-thickness models that recapitulate the structural organization of human skin [127]. Commercial platforms (EpiDerm-FT, MatTek; Phenion FT, Henkel) provide standardized, quality-controlled constructs that enable comparative testing across laboratories. More advanced iterations include immune cell populations (dendritic cells, macrophages) to model inflammatory responses and adipose tissue layers to better represent subcutaneous wound environments [128]. When combined with bacterial inoculation, these infected 3D skin models provide insights into biofilm–tissue interactions, re-epithelialization dynamics, and cytokine responses that monolayer cultures cannot capture. The trade-offs are cost (€200–500 per construct for commercial models), extended culture times (2–4 weeks for full differentiation), and limited throughput that restricts their use to late-stage formulation screening rather than high-throughput optimization [129].

Microfluidic wound-on-a-chip platforms represent the most physiologically sophisticated approach, integrating multiple cell types within perfused microchannels that recapitulate oxygen gradients, shear stress, and spatially organized tissue architecture [130]. These systems can model the wound edge microenvironment, where re-epithelializing keratinocytes interact with underlying fibroblasts and inflammatory infiltrates in the presence of bacterial biofilms. Recent iterations incorporate vascular channels lined with endothelial cells, enabling the study of angiogenesis and immune cell extravasation [131]. For hydrogel evaluation, microfluidic platforms offer the ability to visualize drug release and biofilm disruption in real-time under flow conditions that approximate wound exudate dynamics. The principal barriers to adoption are technical complexity, specialized equipment requirements, and the absence of standardized protocols that would enable cross-laboratory comparisons.

##### Model Selection and Predictive Validity

**Figure 3:**
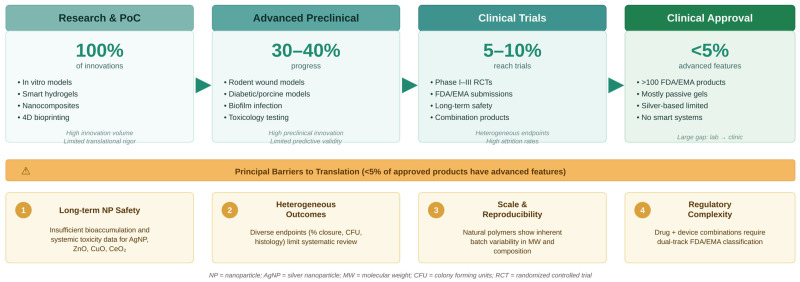
Schematic representation of the translational pathway for hydrogel-based wound dressings, from proof-of-concept research to clinical approval, with estimated attrition rates at each stage (Research/PoC → Advanced Preclinical → Clinical Trials → Clinical Approval). Despite remarkable preclinical innovation, only a small minority of currently approved products incorporate active advanced functionalities beyond passive moisture retention. Principal barriers to translation include: (1) long-term nanoparticle safety data requirements; (2) heterogeneous outcome reporting limiting meta-analytic synthesis; (3) scalability and batch-to-batch reproducibility challenges for natural polymer-based hydrogels; and (4) complex regulatory pathways for combination products (drug + device). Abbreviations: PoC—proof of concept; RCT—randomized controlled trial; FDA—Food and Drug Administration; EMA—European Medicines Agency.

The choice of preclinical model involves fundamental trade-offs between physiological relevance, throughput, cost, and standardization. Simple planktonic assays and crystal violet biofilm quantification remain appropriate for initial screening due to their speed and reproducibility, but positive results at this stage should be interpreted cautiously: they establish minimum activity thresholds rather than clinical potential. The Lubbock model and similar polymicrobial biofilm systems add ecological complexity, but still lack tissue context. Three-dimensional skin constructs and wound-on-chip platforms offer substantially improved predictive validity for wound healing outcomes, but at costs that limit their application to leading candidates. Critically, no in vitro model adequately recapitulates the impaired perfusion, dysregulated immunity, and prolonged chronicity of human diabetic or venous ulcers; even the most sophisticated platforms model acute wound responses over days rather than the months-long stalled healing characteristic of the clinical target. This fundamental gap between any in vitro system and the chronic wound patient population must be acknowledged when interpreting preclinical hydrogel data (Figure 3).

Rodent models are practical, inexpensive, and well-characterized, which is why they dominate the preclinical literature. They are also misleading in important ways: mouse and rat skin heals primarily through wound contraction—the panniculus carnosus muscle beneath the dermis pulls wound edges together—whereas human skin heals through granulation and re-epithelialization. A hydrogel that appears to accelerate wound closure in a mouse may simply be failing to impede the contraction that would happen anyway. Splinted wound models, where a rigid ring is sutured around the wound to prevent contraction, are partially appropriate for this, but introduce their own artifacts (foreign body reaction to the splint, altered wound mechanics, etc.) [132].

Porcine models are the recognized gold standard. Pig skin is structurally similar to human skin: comparable dermal thickness, fixed skin that heals by re-epithelialization rather than contraction, similar appendage density, and wound healing kinetics translate more reliably to clinical outcomes [133]. The trade-off is cost: a single porcine wound healing study costs roughly 5–10 times what an equivalent mouse study costs, requires specialized facilities, and involves ethical considerations that limit sample sizes. This is why porcine data remain scarce for most hydrogel formulations and why the field relies heavily on rodent results that may not translate. A practical consequence is that the majority of hydrogels described in the research literature, including most of the systems discussed in the preceding chapters of this review, have not yet been evaluated beyond rodent models. The number that have reached porcine studies is small. The number with human clinical data are smaller still.

### 5.2. The Current Clinical Landscape

The research literature has seen substantial innovation in nanocomposites, stimuli-responsive materials, and exosome-loaded scaffolds over the past two decades. The current commercial landscape reflects an earlier generation of this work, with most approved products still relying on well-characterized simpler chemistries. Examining why certain technologies have reached patients while others remain in development provides useful guidance for future translational priorities. Of the roughly 100 hydrogel products with FDA and/or EMA clearance, most are simple formulations. Amorphous hydrogels such as Intrasite Gel (Smith & Nephew, Watford, UK) and Purilon Gel (Coloplast, Humlebæk, Denmark) are estimated to account for a substantial share—in the order of half—of market revenue and function as passive moisture donors. They are inexpensive, reliable, and supported by decades of clinical track record, and in terms of core materials chemistry, they are close descendants of the hydrogels developed in the 1980s. Their continued dominance reflects the practical value of simplicity, cost-effectiveness, and consistent performance in routine wound care [134].

The antimicrobial segment is the most dynamic commercially. Aquacel Ag (hydrofiber with ionic silver) is the dominant product in this space, with well-documented clinical evidence supporting its use in critically colonized wounds. Cadexomer iodine products (Iodosorb and Iodoflex, Smith & Nephew, Watford, UK) absorb exudate and release elemental iodine progressively, a controlled-release strategy that predates the current enthusiasm for “smart” drug delivery by several decades and works reliably in practice [135,136].

A few recent products suggest that the commercial sector is beginning to move, cautiously, toward more sophisticated formulations. G4Derm and G4Derm Plus (Gel4Med, Inc., Boston, MA, USA) are FDA-approved peptide-based self-assembling hydrogels that combine antimicrobial function with tissue regeneration support. 3M’s 2025 (Solventum, Maplewood, MN, USA) 2025 line of bioactive impregnated dressings incorporates antimicrobial agents or growth factors into the dressing matrix. These represent incremental advances rather than the kind of leap that the research literature might lead one to expect [137].

The global hydrogel wound care market was valued at approximately $875 million in 2024, with industry projections suggesting growth to around $1.3 billion by the early 2030s [138] [S&S Insider, 2025]. For context, this is a fraction of the overall wound care market (~$20 billion globally), reflecting the fact that foam dressings, alginates, and hydrofibers continue to dominate most clinical wound management protocols [138].

### 5.3. The Translational Gap and Why It Persists

The disconnect between what is published and what is sold is not new, but it is worth examining specifically, because the same barriers keep appearing and the field has not developed convincing strategies to overcome them.

Regulatory complexity: In the United States, a hydrogel that simply retains moisture qualifies as a device under 510 (k) clearance, a relatively fast and inexpensive pathway. The moment a hydrogel incorporates a drug substance (an antibiotic, a growth factor, or a bioactive peptide), it becomes a combination product requiring premarket approval (PMA), typically associated with markedly longer development timelines, often spanning many years and substantially higher development costs that can reach tens of millions of dollars. The FDA’s 2023 proposed rule mandating PMA for dressings containing medically important antimicrobials may further push development away from antibiotic-loaded systems and toward non-antibiotic strategies, which is arguably good policy from a resistance perspective, but constrains commercial options [134]. In Europe, MDR 2017/745 imposes comparably stringent requirements, and the transition from the previous directive has created a bottleneck that has slowed the approval of even conventional wound products [139].

For the advanced systems that dominate the research literature, nanocomposite hydrogels, stimuli-responsive platforms, exosome-loaded scaffolds, the regulatory pathway is not just long, it is largely undefined. Regulatory agencies evaluate products based on precedent, and there is limited precedent for a pH-responsive hydrogel loaded with antimicrobial peptide-decorated silver nanoparticles and stem cell exosomes. Each novel component adds regulatory complexity, testing requirements, and risk.

Manufacturing and scale-up: A hydrogel that works beautifully in a 2 mL batch prepared by a doctoral student does not necessarily work when manufactured in 10,000-unit lots under GMP conditions. Natural polymer variability (batch-to-batch differences in chitosan DDA, alginate M/G ratio, HA molecular weight distribution), multi-step crosslinking procedures, and the need for sterile processing without damaging bioactive payloads all create scale-up challenges. For biological payloads, growth factors, exosomes, live cells, cold chain requirements add logistics costs and limit distribution to markets with reliable cold storage infrastructure, which excludes precisely the low- and middle-income settings where chronic wound burden is highest [140].

These are practical considerations, and they help explain why simpler formulations continue to dominate the approved-product landscape even as the research frontier advances.

Clinical trial design: Even when a hydrogel formulation successfully navigates manufacturing and regulatory hurdles, demonstrating clinical efficacy requires appropriately designed trials, and wound healing trials are notoriously difficult. Chronic wound populations are heterogeneous (different etiologies, comorbidities, wound sizes, locations, infection status), making it hard to achieve adequate statistical power without large sample sizes. Outcome measures are not standardized: some trials report complete wound closure, others report percent area reduction, and others use composite endpoints. Blinding is difficult because hydrogel dressings often look and feel different from comparators. Follow-up periods vary. The result is a clinical evidence base that is growing in volume, but remains difficult to synthesize.

Health economics: The final barrier is financial. Hydrogel dressings are generally more expensive per unit than gauze or simple foam dressings. The economic argument for hydrogels rests on indirect savings: fewer dressing changes (reduced nursing time), lower infection rates (reduced hospitalization costs), and faster healing (shorter overall treatment duration). This argument is plausible, but largely unproven for advanced formulations, because the health economic analyses needed to demonstrate that net cost-effectiveness have not been embedded into the clinical trials that would generate the data. Without this evidence, healthcare purchasers and insurers default to cheaper alternatives.

For next-generation systems with biological payloads (growth factors, exosomes, cells), the cost equation is even more challenging. A cell-laden hydrogel with a shelf life measured in days and a unit cost measured in hundreds or thousands of dollars will need to demonstrate substantial clinical superiority over a $5 tube of Intrasite Gel to justify adoption.

#### The Complexity–Benefit Question

Beyond these barriers lies a more fundamental question that is not always explicitly discussed in the hydrogel literature: whether the added complexity of advanced formulations delivers clinical benefits proportional to their costs. The cost hierarchy spans orders of magnitude, from simple amorphous gels ($5–15/unit) through silver-containing dressings ($15–40/unit) to growth factor-loaded systems ($200–500/treatment) and cell-laden constructs ($1000–5000/application), yet the demonstrated clinical benefit does not always scale proportionally [141,142]. Biological payloads such as growth factors, exosomes, and cells typically require 2–8 °C storage with shelf lives measured in days, which limits distribution to settings with reliable cold chain infrastructure and restricts access in primary care and in low- and middle-income healthcare systems where the chronic wound burden is highest [143]. The argument that higher unit costs are offset by reduced total cost of care through fewer dressing changes, faster healing, and fewer complications is economically plausible, but still requires systematic study, since health economic analyses with prospective data collection are currently available for only a handful of advanced products. For clinical adoption, new technologies generally need to demonstrate either substantially superior outcomes, for example, a clinically meaningful improvement in healing rates, or a lower total cost of care to displace established alternatives, and comparative data of this kind are still accumulating for most advanced hydrogel systems. A chitosan–alginate hydrogel with intrinsic antimicrobial activity that is stable at room temperature and costs around $8 per unit may therefore offer broader global utility than an exosome-loaded nanocomposite costing $500 and requiring cold chain logistics, even where the latter shows superior efficacy in controlled settings. Explicit consideration of the intended clinical setting, acceptable cost range, and logistical constraints from the design stage, rather than at the end of development, would further strengthen the translational value of hydrogel research.

## 6. Conclusions and Future Perspectives

Rather than restating what has been covered in detail in Section 2, Section 3, Section 4 and Section 5, it will be more useful to distinguish between what the current evidence supports with reasonable confidence and what remains aspirational.

Well-supported by clinical and preclinical data: Hydrogels maintain a moist wound environment that favors healing over dry dressing alternatives; this is established beyond reasonable doubt, even if the effect size in clinical trials is more modest than mechanistic arguments might predict. Silver-containing hydrogels and dressings have genuine antimicrobial activity against common wound pathogens, including in biofilm configurations (see Section 3.2), though concerns about cytotoxicity, resistance, and environmental impact constrain their appropriate use to short-term management of critically colonized wounds. Hydrogel encapsulation extends the stability and residence time of labile biomolecules (growth factors, enzymes, peptides) compared to free topical application, a consistent finding across dozens of studies, even if clinical benefit has been demonstrated for very few formulations.

Supported by preclinical data only: Stimuli-responsive drug release (pH, ROS, enzyme-triggered; see Section 3.6), nanocomposite systems for biofilm disruption (Section 4.2), quorum sensing inhibition (Section 4.4), exosome delivery, oxygen-releasing formulations, and ECM-mimetic scaffolds for scarless healing all show encouraging results in laboratory models. None have progressed beyond early-stage clinical evaluation. The gap between in vitro/rodent data and clinical utility is real and should not be minimized.

Largely speculative: AI-driven hydrogel design, 4D bioprinted patient-specific dressings, and microbiome-informed probiotic wound care are scientifically interesting directions for which clinical evidence remains very limited at present. Presenting them as near-term possibilities, as portions of the literature do, may overstate the current state of the field.

The future directions most likely to produce clinical impact within the next decade are not the most scientifically glamorous ones. The three areas below deserve priority.

First, better clinical trials of existing and near-market formulations: The most impactful contribution to the field right now would not be another novel nanocomposite hydrogel tested in mice, but a well-designed, multicenter RCT comparing the best available silver hydrogel (Section 3.2) against a cadexomer iodine dressing (Section 5.2) and a standard-of-care control in patients with biofilm-confirmed diabetic foot ulcers, with standardized endpoints and embedded health economic analysis. The heterogeneous outcome reporting and clinical trial design challenges discussed in Section 5.3 must be addressed for such a trial to generate actionable comparative evidence. A trial of this design and scope is not, to our knowledge, currently available in the published literature. Until comparable evidence is generated, clinical decision-making in this area will continue to depend substantially on expert opinion and indirect comparisons rather than on head-to-head comparative data.

Second, solving manufacturing for the most promising advanced formulations: Among the systems reviewed in this manuscript, growth factor-loaded hydrogels and enzyme-mediated biofilm dispersal systems (see Section 4.2 for lysostaphin, dispersin B, and DNase I approaches) are arguably closest to translational readiness, the active agents are well characterized, the biological mechanisms are validated, and the main barriers are formulation stability, manufacturing reproducibility, and cost. The crosslinking strategies discussed in Section 2.5, particularly enzymatic crosslinking methods that avoid cytotoxic residues, may offer advantages for these biologically active formulations. The scale-up and regulatory challenges outlined in Section 5.3 remain the primary obstacles to clinical translation. Focused investment in process engineering for these specific systems would yield faster clinical returns than continued diversification into ever more complex multi-component platforms.

Third, designing for accessibility: Chronic wounds disproportionately affect elderly, diabetic, and socioeconomically disadvantaged populations, precisely the groups with least access to expensive, cold-chain-dependent advanced therapies. Hydrogel formulations based on abundant biopolymers, chitosan, cellulose and alginate, with shelf-stable lyophilized formats that reconstitute with sterile water, offer a path to advanced wound care that does not depend on high-income healthcare infrastructure. The intrinsic antimicrobial activity of chitosan, despite its pH-dependent limitations previously discussed, makes it particularly attractive for low-resource settings where cold-chain storage for more sophisticated antimicrobial payloads is unavailable. This is not just an equity argument; it is where the largest unmet clinical need exists and where the largest patient populations could benefit.

The hydrogel wound care field has no shortage of innovative materials (Section 2) or clever biological strategies for antimicrobial delivery (Section 3) and biofilm management (Section 4). What it lacks is the disciplined translational work, rigorous testing models (Section 5.1), adequately powered trials, manufacturing solutions, and cost-conscious design (Section 5.3), which are needed to convert that innovation into treatments that reach the patients who need them.

In summary, three key messages emerge from this review. First, biofilm must be recognized as a distinct therapeutic target requiring dedicated disruption strategies, not merely higher antimicrobial doses. Second, the gap between preclinical promise and clinical reality is not primarily a materials science problem, it is a translational infrastructure problem encompassing model validity, manufacturing scalability, regulatory pathways, and health economic evidence. Third, the patients most likely to benefit from advanced hydrogel wound care are precisely those least likely to access it under current development paradigms; designing for global accessibility should be a priority, not an afterthought. Addressing these challenges will require closer collaboration between materials scientists, wound care clinicians, regulatory experts, and health economists than the field has historically achieved.

## Figures and Tables

**Table 1:** Comparative analysis of polymer systems for antimicrobial wound care hydrogels.

Parameter	Chitosan	Alginate	Hyaluronic Acid	Bacterial Cellulose	Collagen/Gelatin	PVA	PEG
Intrinsic antimicrobial activity	Yes (pH-dependent)	None	None	None	None	None	None
Drug loading capacity	Moderate (electrostatic)	High (ionic)	Moderate	High (physical)	Moderate	High	High (tunable)
Release control	Limited (burst-prone)	Poor (dissolution)	Moderate	Moderate	Poor (MMP degradation)	Good (physical)	Excellent (tunable)
Biofilm penetration potential	Moderate (cationic)	Low	Low	Low	Low	Low	Tunable (with NPs)
Mechanical strength	Poor (brittle)	Poor	Moderate	Good	Poor-Moderate	Good	Moderate-Good
Batch reproducibility	Variable (DDA, MW)	Variable (M/G)	Variable (MW)	Moderate	Variable	Excellent	Excellent
Manufacturing complexity	Low-Moderate	Low	Moderate-High	High (fermentation)	Moderate	Low	High
Regulatory precedent	Limited	Established	Limited	Limited	Established	Established	Limited
Clinical readiness (TRL)	3–4	7–9	4–5	3–4	6–7	7–8	4–5

Abbreviations: DDA—degree of deacetylation; MW—molecular weight; M/G—mannuronic/guluronic acid ratio; MMP—matrix metalloproteinase; NP—nanoparticle; TRL—Technology Readiness Level (1–9 scale).

## Data Availability

Not applicable.

## Abbreviations

The following abbreviations are used in this manuscript:

**Abbreviation**	**Definition**
AgNPs	Silver nanoparticles
AHL	Acyl-homoserine lactone
AMP	Antimicrobial peptide
BC	Bacterial cellulose
CHG	Chlorhexidine
CIP	Ciprofloxacin
Cu-BG	Copper-doped bioactive glass
CuO	Copper oxide
CeO_2_	Cerium oxide
DDA	Degree of deacetylation
DTP	Dithiobis(propanoic dihydrazide)
ECM	Extracellular matrix
EDC/NHS	1-Ethyl-3-(3-dimethylaminopropyl)carbodiimide/N-hydroxysuccinimide
EMA	European Medicines Agency
EPS	Extracellular polymeric substance
FDA	Food and Drug Administration
FIC	Fractional inhibitory concentration
GelMA	Gelatin methacrylate
GEN	Gentamicin
GMP	Good manufacturing practice
HA	Hyaluronic acid
HBPL	Hyperbranched poly-L-lysine
HRP	Horseradish peroxidase
IPN	Interpenetrating polymer network
LCST	Lower critical solution temperature
MDR	Multidrug resistant
MIC	Minimum inhibitory concentration
MMP	Matrix metalloproteinase
MRSA	Methicillin-resistant *Staphylococcus aureus*
MSC	Mesenchymal stem cell
MTT	3-(4,5-Dimethylthiazol-2-yl)-2,5-diphenyltetrazolium bromide
PAA	Poly(acrylic acid)
PEG	Poly(ethylene glycol)
PLGA	Poly(lactic-co-glycolic acid)
PMA	Premarket approval
PMPC	Poly(2-methacryloyloxyethyl phosphorylcholine)
PNIPAAm	Poly(N-isopropylacrylamide)
PSBMA	Poly(sulfobetaine methacrylate)
PVA	Poly(vinyl alcohol)
PVP-I	Povidone-iodine
QS	Quorum sensing
RGD	Arginine-glycine-aspartate (peptide motif)
ROS	Reactive oxygen species
TEMPO	2,2,6,6-Tetramethylpiperidine-1-oxyl
UV	Ultraviolet
VEGF	Vascular endothelial growth factor
ZnO	Zinc oxide
